# CircRNAs in hepatocellular carcinoma: characteristic, functions and clinical significance

**DOI:** 10.7150/ijms.74713

**Published:** 2022-11-14

**Authors:** Yujun Zhou, Xingkang Mao, Rui Peng, Dousheng Bai

**Affiliations:** 1 Department of Hepatobiliary and Pancreatic Surgery, Huaihua First People's Hospital, Huaihua, Hunan, P. R. China.; 2 Cardiovascular Center, Huizhou First Municipal People's Hospital, Huizhou, Guangdong, P. R. China.; 3 Department of Hepatobiliary Surgery, Clinical Medical College, Yangzhou University, Yangzhou, Jiangsu, P. R. China.

**Keywords:** hepatocellular carcinoma, circRNAs, molecular sponge, circRNA-miRNA-mRNA axis

## Abstract

Hepatocellular carcinoma (HCC) is one of the most common and serious types of cancer worldwide, with high incidence and mortality rates. Circular RNAs (circRNAs) are a novel class of non-coding RNA with important biological functions. In recent years, multiple circRNAs have been found to be involved in the biological processes of tumorigenesis and tumor development. Increasing evidence has shown that circRNAs also play a crucial role in the occurrence and development of HCC. However, the specific molecular mechanism of circRNAs in HCC has not been fully elucidated. The present review systematically summarized the classification and basic characteristics of circRNAs, their biological functions and their role in the occurrence and development of HCC. By summarizing the previous studies on circRNAs in HCC, this study aimed to indicate potential approaches to improving the early diagnosis and treatment of HCC.

## Introduction

Liver cancer is one of the most common and serious types of cancer worldwide, with high incidence and mortality rates 1. Hepatocellular carcinoma (HCC) accounts for 90% of all primary liver cancer cases and is the most common histological subtype 2. Currently, treatment options for HCC include surgical resection, liver transplantation, image-guided ablation, transcatheter arterial chemoembolization and targeted therapy, such as sorafenib, lenvatinib and regorafenib 3-5. However, despite these comprehensive treatments, the prognosis of HCC remains poor 6. Therefore, exploring the pathogenesis of HCC, identifying new therapeutic targets and designing more effective treatments are crucial.

Circular RNAs (circRNAs) are a circular type of non-coding RNAs (ncRNAs) with important biological functions that are characterized by a covalently closed ring structure without a 5' cap and 3' polyadenylation tail 7. It has been shown that circRNAs are involved in the pathogenesis of a variety of human diseases, including cardiovascular diseases, diabetes, neurological diseases and cancer 8. An amount of evidence indicates that circRNAs play a key role in the occurrence and development of HCC. Some circRNAs can promote the progression of HCC. For example, circ_0008450 can promote the proliferation, invasion and migration of HCC cells and inhibit apoptosis by regulating miR-548p 9; circRNA-104718 can also promote the proliferation, invasion, and proliferation of HCC cells by regulating the miRNA-218-5p/TXNDC5 axis. migration and inhibit the apoptosis of HCC 10. However, some circRNAs have inhibitory effects on the progression of HCC. For example, circADAMTS14 inhibits the proliferation, invasion and migration of HCC cells and promotes HCC apoptosis by regulating miR-572/RCAN1 11; circRNA-5692 exerts the same inhibitory effect by regulating miR-328-5p/DAB2IP 12. Although a small number of circRNAs have been functionally characterized, the role of circRNAs in the occurrence and development of HCC has not been fully elucidated and requires further investigation.

The role of circRNAs in the occurrence and development of HCC should be further studied. Therefore, the present study described the basic characteristics of circRNAs, elaborated on their biological functions and summarized their specific roles in HCC, to provide basic ideas for the further study of circRNAs in HCC.

## Basic characteristics and classification of circRNAs

As an important member of ncRNA, circRNAs have been widely studied, and have been shown to have the following basic characteristics: 1. CircRNAs have a covalent closed continuous loop, which means that they do not have a 5'-3' polar or polyadenylated tail. This feature allows circRNAs to escape the fate of being degraded by exonuclease, and theoretically makes them have a more stable structure than linear RNAs 13; 2. the circRNA studies that have been carried out so far have indicated that circRNAs are widespread in eukaryotic cells, and consist of several types 14; 3. the distribution of circRNAs in cells is not uniform, as most of them are located in the cytoplasm and a few in the nucleus 15; 4. the majority of circRNA sequences are highly conserved within a species 16; 5. CircRNAs are often expressed in different developmental stages and various tissues with significant specificity 17; 6. CircRNAs play a regulatory role at the level of transcription or post-transcription 15.

**Figure 1:**
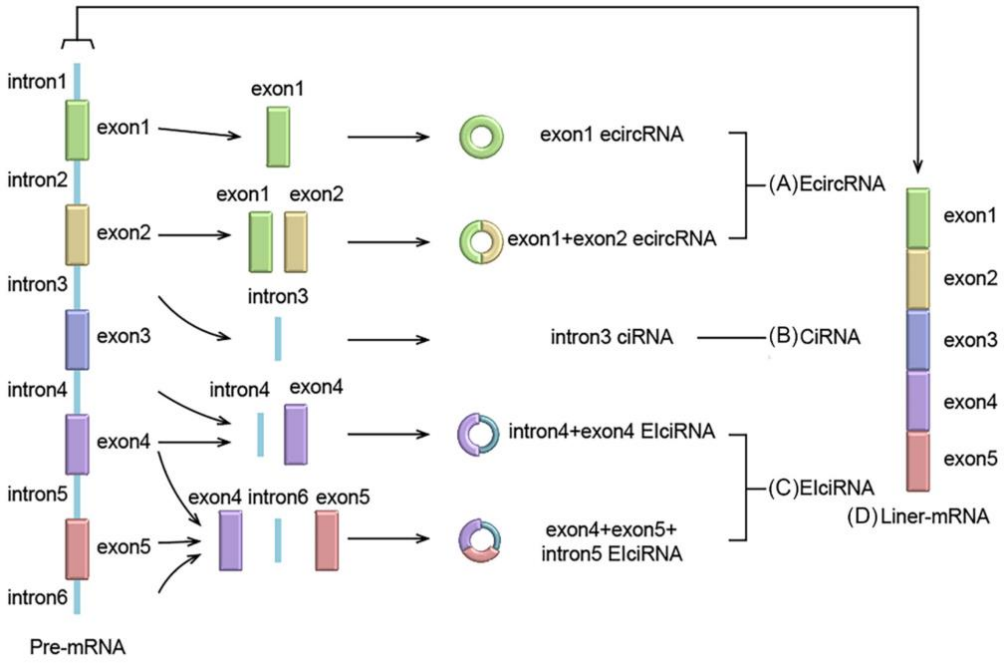
Formation of circRNA and linear RNA. **(A)** Exon-circRNA (EcircRNA): EcircRNAs are only derived from exons; **(B)** Circular intron RNA (CiRNA): CiRNAs are derived from intron lariats; **(C)** Exon-intron circRNA (EIciRNA): EIciRNA are derived from both exons and intron lariats; **(D)** Traditional linear mRNA comes from pre-mRNA classic splicing.

Pre-mRNA is the parent of both circRNAs and linear RNA. Unlike the classical splicing of linear RNA, most circRNAs are formed by reverse splicing of pre-mRNA 18. circRNAs have a wide range of sources, and circRNAs may be produced in all regions of the genome, including intergenic, intronic, antisense and untranslated regions 15. circRNAs are classified into the following three categories, based on origin: Exon circRNAs (EcircRNAs), circular intron RNAs (CiRNAs) and exon-intron circRNAs (EIciRNAs). circRNAs are divided into these three subtypes due to their different cyclization mechanisms 19. EcircRNAs are derived from exons, and most of the circRNAs currently studied are EcircRNAs. According to the different formation methods of EcircRNAs, two different models have been proposed. The first is the lariat-driven circularization model, which contains only one exon, and is formed by reverse-splicing the 5' splice site of the same exon to the 3' splice site. The second is the circularization model driven by intron pairs, which contains multiple exons and is formed by reverse splicing of the 5' splice site to the 3' splice site of another exon. CiRNAs, unlike EcircRNAs, lack exons and are all produced from intron lariats, which can avoid the debranching and degradation of normal introns. EIciRNAs integrate the characteristics of EcircRNAs and CiRNAs, including both exons and introns that are not completely spliced. And through different combinations, various types of EIciRNAs can be formed (Figure 1).

CircRNAs have been shown to play an important role in a variety of physiological and pathological processes, including tumorigenesis and tumor progression. Understanding and clarifying these basic characteristics is important to further study the function and mechanism of circRNAs in HCC.

## The biological function of circRNAs

**Figure 2:**
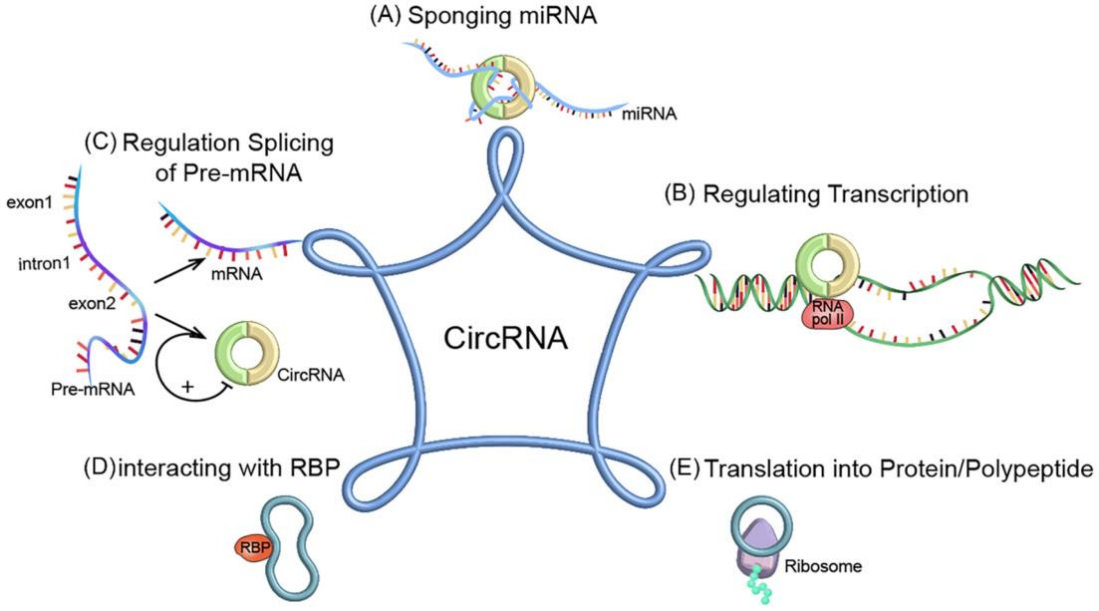
** The functional mechanisms of circRNAs. (A)** Acting as miRNA sponge; **(B)** Regulating transcription;** (C)** Regulating splicing of pre-mRNA; **(D)** Interacting with RBP; **(E)** Translated into protein/polypeptide.

CircRNAs play a key role in cancer growth, metastasis and treatment resistance. The biological functions of circRNAs have been extensively studied. Their functions can be divided into five categories: (1) Acting as microRNA (miRNA) sponges to inhibit miRNA-related functions 20; (2) regulating transcription and translation; (3) regulating alternative splicing of pre-mRNAs; (4) regulating gene expression by interacting with RNA-binding proteins (RBPs); (5) some circRNAs can be translated into proteins as translation templates (Figure 2).

### circRNAs can function as miRNA sponges

According to the central dogma of genetics, mRNAs can be translated into proteins, which comprises the dominant protein formation process 21. miRNAs are a type of endogenous RNA ~22 nt in length, which can play an important regulatory role by targeting mRNAs for cleavage or translation inhibition 22. The discovery of miRNA response elements shows that circRNAs can competitively bind to miRNAs to affect the pathophysiological process of animals and plants. This mechanism is widespread in the human body and plays a regulatory role in several biological processes. CircRNAs can be used as a miRNA molecular sponge to prevent miRNAs from performing their original biological function 23, or they can competitively inhibit miRNAs by binding to the target molecule. This results in the miRNA function being inhibited, which, in turn, affects the downstream function. One of the best examples of this circRNA mechanism is cerebellar degeneration-related protein 1 antisense (CDR1as). First, CDR1as can bind to miR-7, since it has >70 selectively conserved miR-7 binding site circular molecules 24. CDR1as can also be used as a molecular sponge to bind to miR-1270 25, miR-135-5p 26, miR-219a-5p 27 and miR-671-5p 28. CDR1as has been closely associated with the occurrence and development of various cancer types, such as HCC 29-31, gastric 32, colorectal 33 and ovarian 26 cancers. Similar molecular sponging mechanisms include circ-CDYL 34, circASAP1 35, circPVT1 36, 37 and cSMARCA5 38. In conclusion, the circRNA-miRNA axis may play an important role in the development of cancer. Further research on the direct mechanism of the circRNA-miRNA axis is required to fully elucidate the code of life.

### circRNAs can act as transcriptional regulators

Unlike EcircRNAs, which are mainly distributed in the cytoplasm, CiRNAs and EIciRNAs retain introns and are largely located in the nucleus. The mechanism of action of these two circRNAs is also different from that of EcircRNAs. CiRNAs and EIciRNAs cannot be used as molecular sponges to participate in the pathophysiology of organisms, but they can be combined with RNA polymerase II to affect the transcription process of the parent gene, thus also affecting protein synthesis 39. EIciRNAs can participate in the regulation of the transcription mechanism, since its sequence retains the intron of the parent gene. The following describes several typical adjustment procedures: circ-EIF3J and circ-PAIP2 contain the sequence of the promoter region of the parent gene, so they can interact with U1 snRNPs and RNA polymerase II in the promoter region of the host gene, thereby enhancing the transcription process of the parent genes (PAIP2 and EIF3J) 40. CiRNAs can also affect the activity of RNA polymerase II, thereby regulating the transcription process, further affecting the amount of target mRNA. This mechanism corresponds to CiRNAs such as ci-ANKRD52, ci-MCM5 and ci-SIRT7, all of which extend the activity of RNA polymerase II to promote transcription, thereby promoting the expression of parental genes. On the contrary, when the levels of these CiRNAs are reduced, the transcription levels of their parent genes are reduced 41.

### CircRNAs can compete with traditional linear mRNAs for pre-mRNAs

In the source-based classification, exon-derived circRNAs constitute the majority 42. In addition, both circRNAs and traditional linear mRNAs are derived from pre-mRNAs. Pre-mRNAs form circRNAs through exon circularization and can compete with linear mRNAs through canonical splicing. Therefore, circRNAs formed by exon circularization can compete with linear mRNAs in this splicing process 43. Pre-mRNA exon circularization for the formation of circRNAs inevitably leads to the existence of fewer pre-mRNAs for the formation of mRNAs through canonical splicing. This suggests that the formation of circRNAs usually occurs at the expense of reduced mRNA production 44. Similarly, the canonical spliceosome mechanism can affect the formation of circRNAs from pre-mRNAs 45. Consequently, the production of circRNAs can block pre-mRNA splicing into mRNAs 46, 47. As a result, the production of circRNAs leads to the reduction of its linear isoform mRNA 48, which leads to the production of circRNAs blocking the process of mRNA formation from pre-mRNAs, ultimately affecting protein production. However, this mechanism of competing for splicing sites requires further systematic research to provide data support, as it cannot fully account for the effect of this mechanism on the final product.

### CircRNAs can regulate gene expression by interacting with RBPs

Post-transcriptional regulation is not limited to the splicing process; RBPs also play an important role in it. circRNAs can bind to miRNAs to act as miRNA molecular sponges, while certain circRNAs are also able to bind to RBPs to act as a protein sponge, thus affecting the post-transcriptional modification process, which all possess RBPs binding sites. For example, the human antigen R (HuR) can bind to PABPN1 mRNA and positively regulate the translation process of PABPN1. circPABPN1 acts as an RBPs sponge. It can first bind to HuR and inhibit the binding of HuR to PABPN1 mRNA, thereby inhibiting the positive effect of HuR on the translation of PABPN1 mRNA 49. This mechanism of acting as an RBP molecular sponge has also been observed in circ-FOXO3 50, circ-MBL 43 and circ-Amotl1 51-53.

### circRNAs possess a translational ability

Due to the lack of the polyadenylated tails and internal ribosome entry sites (IRES) required for protein translation, circRNAs are widely classified as endogenous non-coding RNAs (ncRNAs) 54, 55. However, the latest research appears to have proven that certain circRNAs have the ability to be translated into proteins. At present, 4 such circRNAs have been discovered: circ-ZNF609 56, circ-SHPRH 57, circ-FBXW7 58 and circMBL 59. When IRES is introduced into the circRNA sequence, circRNAs can be translated *in vivo* and *in vitro*. However, it remains unclear whether such a reaction can occur in an organism, or whether it can be widespread and participate in the pathophysiology of the human body. This type of biological behavior undermines our understanding of circRNAs. Therefore, it is worth studying whether circRNAs can be considered non-coding RNAs.

In recent years, there has been an increasing amount of research results on the biological functions of circRNAs, a considerable portion of which are different from our current understanding. circRNAs are an important supplement to the central dogma of genetics. They play a regulatory role in multiple steps of protein synthesis and participate in the pathophysiological process of tumor development. However, our understanding of circRNAs remains incomplete, and further research is required.

## Functional circRNAs in HCC

CircRNAs are commonly aberrantly expressed in tissues from several types of cancer, including HCC. These differentially expressed circRNAs ultimately promote or inhibit the occurrence and development of cancer. These circRNAs may play a crucial role in the carcinogenesis and development of HCC.

### CircRNAs function as oncogenes in HCC

In recent studies, several circRNAs have been found to be significantly overexpressed in HCC, as compared with adjacent tissues, and it has been suggested that they may play an oncogenic role in the development of HCC. The upregulated circRNAs in HCC are listed in Table 1.

Although different circRNAs regulate HCC in different ways, recent studies have found that different circRNAs may regulate HCC through a common pathway 34, 35, 60-62. It has been reported that the expression of circ-CDYL in HCC tissue is higher than that in paracancerous tissue 34. The high expression of circ-CDYL has been found to significantly promote the formation of HCC 34. Functional experiments have shown that circ-CDYL could act as a molecular sponge of miR-892a and miR-328-3p, and inhibit their binding to HDGF and HIF1AN, thereby promoting the expression of HDGF and HIF1AN 34. This, in turn, has been shown to lead to the activation of the PI3K-AKT-mTORC1/β-catenin and NOTCH2 pathways, promoting the expression of the effector proteins BIRC5/SURVIVIN and MYC proto-oncogene 34. In conclusion, the expression of circ-CDYL is increased in the early stage of HCC and promotes the formation of HCC through a series of gene regulations 34. In addition, hsa_circ_0046600 can also regulate hypoxia-inducible factor-1α (HIF-1α) through sponging miR-640. Recent studies have shown hsa_circ_0046600 to be significantly upregulated in HCC tissues, as compared with adjacent normal tissues, and the expression of hsa_circ_0046600 to be significantly correlated with clinicopathological factors 60. In functional experiments, the downregulation of hsa_circ_0046600 was found to significantly inhibit the migration of HCC cells. Mechanistically, hsa_circ_0046600 is mainly used as a molecular sponge of miR-640 to regulate the level of HIF-1α, thereby regulating the biological behavior of HCC. In conclusion, hsa_circ_0046600 can promote the migration of HCC cells through the miR-640/node HIF-1α axis 60.

As compared with adjacent tissues, circASAP1 is highly expressed in HCC tissues. Functional experiments have shown that the overexpression of circASAP1 can promote cell proliferation, migration and invasion 35. circASAP1 can promote the expression of mitogen-activated protein kinase (MAPK)1 and colony-stimulating factor (CSF)-1 by sponging miR-326 and miR-532-5p. In conclusion, circASAP1 can regulate the miR-326/miR-532-5p-MAPK1/CSF-1 axis, thereby regulating the biological behavior of HCC 35. Hsa_circ_0101432 is significantly upregulated in HCC. The upregulated hsa_circ_0101432 can enhance the proliferation and invasion ability of HCC cells and inhibit apoptosis. Mechanistically, hsa_circ_0101432 has been reported to act as a molecular sponge of miR-1258 and miR-622 to enhance the expression of MAPK1 to promote tumor growth. In conclusion, hsa_circ_0101432 inhibits HCC cell apoptosis by targeting miR-1258 and miR-622 and upregulating MAPK1 mRNA expression, and promoting cell proliferation, invasion and HCC tumor growth 61. The expression of circMAN2B2 was reported by Fu et al. 62 to be higher in HCC tissue than that in paracancerous tissue. It was also reported to act as a molecular sponge of miR-217 to regulate the expression of MAPK1, thereby promoting cell proliferation.

Although different circRNAs regulate HCC in different ways, recent studies have found that the same circRNAs can also regulate HCC in different ways 9, 36, 37, 63-65. CircPVT1 is highly expressed in HCC, which indicates a poor prognosis. Functional experiments have shown that the downregulation of circPVT1 can reduce the proliferation and migration capacity of HCC cells, whereas the upregulation of circPVT1 can promote the growth and migration of HCC cells. In terms of mechanism, circPVT1 can directly bind with miR-203, and promote the occurrence and development of HCC by regulating the miR-203/homebox D3 (HOXD3) pathway. In conclusion, circPVT1 can regulate the progression of HCC by adjusting the miR-203/HOXD3 axis 37. The circPVT1 expression is significantly upregulated in HCC tissues and cell lines. The downregulation of circPVT1 can significantly reduce the proliferation of HCC cells and increase apoptosis. Mechanistically, circPVT1 can be used as a molecular sponge to adsorb miR-3666 and reduce the inhibitory effect of miR-3666 on SIRT7. In conclusion, the circPVT1/miR-3666/SIRT7 axis has been shown to play an important role in the occurrence and development of HCC 36.

It was reported by Huang *et al* 63 that the activity of the mTOR signaling pathway can be regulated by circRNA-100338. Further research found that circRNA-100338 regulates the mTOR signaling pathway through the circRNA-100338/miR-141-3p/RHEB axis, and may be used as an important indicator to predict the prognosis of HCC patients 64. The expression of circRNA_100338 in HCC tissues is higher than that in adjacent tissues, indicating a poor prognosis. Bioinformatics analysis and luciferase experiments have confirmed that circRNA_100338 regulates the occurrence and development of HCC through binding to miR-141-3p, suggesting that circRNA_100338 is a potentially valuable biomarker for HCC diagnosis and treatment 64.

By measuring the content of hsa_circ_0008450 in HCC tissues and cells, Lin *et al* found that hsa_circ_0008450 was significantly increased. Functional experiments have suggested that downregulating hsa_circ_0008450 can significantly inhibit cell proliferation, invasion and migration. Mechanistically, hsa_circ_0008450 has been found to promote the enhancer of zeste homolog 2 (EZH2) protein expression by sponging miR-214-3p. That study demonstrated that the hsa_circ_0008450/miR-214-3p/EZH2 axis plays an oncogenic role in the occurrence of HCC and may serve as a new target for the treatment of HCC 65. In HCC tissue specimens and cell lines, Zhang *et al* also found that the circ_0008450 expression was upregulated, which was associated with poor prognosis 9. Functional tests have suggested that the downregulation of circ_0008450 can inhibit the proliferation, migration and invasion of HCC cells, while increasing apoptosis. Mechanistically, circ_0008450 has been proven to be a sponge of miR-548p, and circ_0008450 to regulate the process of HCC by combining with miR-548p 9.

CircRNAs are abundant in cells and tissues, and recent studies have shown that exosomes also contain large amounts of circRNAs. circRNAs in exosomes have also been found to be key regulators in the occurrence and development of HCC. It has been reported that circPTGR1 is upregulated in serum exosomes of HCC patients and is associated with clinical stage and prognosis. circPTGR1 can be used as a molecular sponge of miR-449a and reduce the inhibitory effect of miR-499a on hepatocyte growth factor receptor (MET)-related mRNA, thereby promoting MET protein translation and HCC cell migration and invasion 66. Exosomes also contain a large amount of circ-deubiquitination (circ-DB). Research has shown that the exosomal circ-DB level increases as the body fat proportion increases. It has been reported by functional experiments that exosomal circ-DB may not only promote HCC growth, but also reduce DNA damage. Mechanistically, exosomal circ-DB can act as a molecular sponge to adsorb miR-34a, thereby reducing the inhibitory effect of miR-34a on DB-related USP7 67. As compared with normal paracancerous tissues and normal liver cell lines, the expression of circRNA Cdr1as in HCC cell lines and tissues was significantly higher. In functional experiments, the upregulation of circRNA Cdr1as can promote the proliferation and migration of HCC cells. Mechanistically, circRNA Cdr1as promotes the protein expression of AFP by competitively binding to miR-1270 in HCC. Further research has shown that exosomes also contain a large amount of circRNA Cdr1as, the overexpression of which in HCC cells promotes the proliferation and migration of surrounding normal cells. In conclusion, circRNA Cdr1as can promote the protein expression of AFP by binding to miR-1270, thereby promoting the proliferation and migration of HCC cells 68.

Several other circRNAs 10, 69-97 have been identified to play an oncogenic role in the development and progression of HCC, and they are listed in Table 1. These circRNAs are highly expressed in HCC and act as oncogenes by promoting cancer cell proliferation, invasion, migration, anti-apoptosis, drug resistance and immune escape. Different circRNAs may regulate HCC through a common pathway, and the same circRNAs can also regulate HCC in different ways. HCC cells can not only regulate the occurrence and development of HCC through the differential expression of circRNAs in them, but also distant cells and tissues through exosomes containing differentially expressed circRNAs.

### CircRNAs may function as tumor suppressors in HCC

In addition to the aforementioned circRNAs, some other circRNAs exhibit a significantly lower expression in HCC than adjacent tissues. It has been suggested that they may play the role of tumor suppressor genes in the development of HCC. These downregulated circRNAs in HCC are listed in Table 2.

The expression of cSMARCA5 is lower in HCC tissues, as compared with that in adjacent tissues, which indicates a poor prognosis. Functional experiments have suggested that cSMARCA5 can inhibit the proliferation and migration of HCC cells. Mechanistically, cSMARCA5 can promote the expression of TIMP3 by sponging miR-17-3p and miR-181b-5p. TIMP3 is a well-known tumor suppressor gene that can significantly inhibit the occurrence and development of tumors. In conclusion, the cSMARCA5/miR-17-3p/miR-181b-5p/TIMP3 axis plays an important role in the proliferation and migration of HCC 38.

Recent studies have shown that circTRIM33-12 is downregulated in HCC tissues and cell lines, which indicates a poor prognosis in HCC patients. Functional experiments have suggested that the downregulation of circTRIM33-12 in HCC cells increases their proliferation, migration, invasion and immune escape. Mechanistically, circTRIM33-12 can be used as a molecular sponge to bind miR-191, reducing the inhibitory effect of miR-191 on TET1. The upregulation of TET1 further leads to a marked reduction in the 5-hydroxymethylcytosine (5hmC) level. In conclusion, the circTRIM33-12/miR-191/TET1/5hmC axis plays an important role in the occurrence and development of HCC 98.

CircMTO1 has also been reported as hsa_circRNA_0007874 or hsa_circRNA_104135 and is significantly downregulated in HCC tissues. Poor prognosis in HCC patients was found to be associated with a low circMTO1 expression. Through functional experiments, the downregulation of circMTO1 can significantly enhance the proliferation and invasion ability of HCC cells. Mechanistically, circMTO1 has been shown to act as a molecular sponge that binds to miR-9 to reduce the inhibitory effect of miR-9 on p21, thereby promoting p21 expression. In conclusion, the circMTO1/miR-9/p21 axis plays an important role in the progression of HCC, suggesting that circMTO1 may be a potential target for the treatment of HCC 99.

Studies have shown that circADAMTS13 is significantly downregulated in HCC organizations. In addition, clinical pathological analysis has suggested that the upregulation of circADAMTS13 is negatively correlated with tumor size, but positively correlated with prognosis. Functional experiments have reported that the overexpression of circADAMTS13 can significantly inhibit the proliferation of HCC cells. Bioinformatics analysis and luciferase reporter gene detection revealed that circADAMTS13 can be combined with miR-484 as a molecular sponge. Therefore, these results indicated that circADAMTS13 can act as a tumor suppressor in the process of HCC through the functional pathway of sponging miR-484 100. As compared with normal paracancerous tissues and normal liver cell lines, the expression of circADAMTS14 in HCC cell lines and tissues was significantly lower. As shown by functional experiments, the upregulation of circADAMTS14 can induce HCC cell apoptosis and inhibit cell proliferation and invasion. Mechanistically, circADAMTS14 promotes the protein expression of regulator of calcineurin 1 (RCAN1) by competitively binding to miR-572 in HCC. In conclusion, circADAMTS14 can suppress the HCC process by adjusting the miR-572/RCAN1 axis 11.

Several other circRNAs 12, 101-110, which act as tumor suppressor genes in the development and progression of HCC, have been listed in Table 2. These circRNAs have been reported to act as tumor suppressor genes. In the occurrence and development of HCC, these circRNAs inhibit the proliferation, invasion, migration, anti-apoptosis, drug resistance and immune escape of cancer cells. Research on circRNAs in HCC remains highly insufficient; therefore, further research on the role of circRNAs in HCC is required.

Medical history, symptoms, signs, laboratory examinations and imaging examinations have markedly improved the diagnosis of HCC. However, several patients are already at an advanced stage at diagnosis, which prevents them from benefiting from surgery. Liver puncture cytology biopsy is the gold standard for diagnosing HCC; however, this invasive operation is difficult for patients to accept, and penetrating the tumor tissue may not be possible, depending on the experience of the clinician. The abnormal expression of circRNAs in HCC tissues and cells highlights their potential value as new diagnostic biomarkers. Exosomes, in particular, also contain multiple circRNAs. circRNAs may be used as biomarkers for early HCC diagnosis or prediction of prognosis. In addition, the aforementioned studies indicate that circRNAs play an important role in the proliferation, invasion, migration, anti-apoptosis and drug resistance of HCC cells; therefore, circRNAs may also serve as potential therapeutic targets in HCC.

## Conclusions

HCC is one of the most common types of cancer and highly detrimental to people's health; however, its molecular mechanisms are incompletely understood. circRNAs are an important class of non-coding RNAs. Emerging evidence indicates that circRNAs are correlated with the pathogenesis of various human diseases. Furthermore, circRNAs have been shown to play important roles in the occurrence and development of HCC. First, circRNAs can affect several biological processes of HCC, including cell proliferation, migration, invasion, apoptosis and drug resistance. Second, circRNAs may be used as biomarkers to diagnose and evaluate the prognosis of patients with HCC, which may markedly improve early HCC diagnosis and treatment. Finally, circRNAs may also serve as potential targets for HCC treatment.

CircRNAs are an emerging class of non-coding RNAs that act as endogenous competitive RNAs in the human body. Although research on circRNAs remains insufficient, we believe that circRNAs may be widely used for the diagnosis, treatment and prognosis assessment of HCC, thereby improving the prognosis of patients with HCC. However, further research is required to confirm their value in the clinical setting.

## Figures and Tables

**Table 1:** The up-regulated circRNAs in HCC

CircRNA	Gene Symbol	Expression Change	miRNA sponged	Competitive mRNA	Function	Reference
circ-CDYL	CDYL	Up	miR-892a miR-328-3p	HDGF HIF1AN	proliferation (+); T-ICs (+); spheroid growth (+); chemotherapy resistance (+); EPCAM^+^cells (+)	34
circ-0046600		Up	miR-640	HIF-1α	migration (+);	60
CircASAP1	ASAP1	Up	miR-326; miR-532-5p	MAPK1; CSF-1	proliferation (+); migration (+); invasion (+)	35
hsa_circ_0101432	RPPH1	Up	miR-1258/miR-622	MAPK1	proliferation (+); invasion (+); apoptosis (-)	61
circMAN2B2	MAN2B2	Up	miRNA-217	MAPK1	proliferation (+)	62
circPTGR1	PTGR1	Up	miR-449a	MET	migration (+); invasion (+)	66
circ-DB		Up	miR-34a	USP7/Cyclin A2		67
circRNA Cdr1as	CDR1	Up	miR-1270	AFP	proliferation (+); migration (+)	68
circRNA PVT1	PVT1	Up	miR-203	HOXD3	proliferation (+); migration (+)	36
circPVT1	PVT1	Up	miR-3666	SIRT7	proliferation (+); apoptosis (-)	37
circRNA-100338		Up	miR-141-3p	RHEB/EIF5; mTOR signaling pathway	proliferation (+)	63
circRNA-100338	SNX27	Up	miR-141-3p		migration (+); invasion (+)	64
hsa_circ_0008450	CMTM3	Up	miR-214-3p	EZH2	proliferation (+); migration (+); apoptosis (-)	65
circ_0008450	CMTM3	Up	miR‐548p		proliferation (+); migration (+); invasion (+); apoptosis (-)	9
hsa_circRNA_103809	ZFR	Up	miR-377-3p	FGFR1/ERK	proliferation (+); cell cycle (+); migration (+)	72
circRNA-104718		Up	miRNA-218-5p	TXNDC5	proliferation (+); migration (+); invasion (+); apoptosis (-)	10
circMYLK	MYLK	Up	miR-362-3p	Rab23	proliferation (+); invasion (+); migration (+)	71
circ-ZNF652	ZNF652	Up	miR-203/ miR-502-5p	Snail	Invasion (+); migration (+)	73
circ_0000267		Up	miR‐646		proliferation (+); migration (+); invasion (+); apoptosis (-)	74
circ-FOXP1	FOXP1	Up	miR-875-3p/ miR-421	SOX9	proliferation(+);invasion(+); apoptosis (-)	69
circRNA_104075		Up	miR-582-3p	YAP		75
hsa_circ_0078710	THBS2	Up	miR-31	HDAC/CDK2/cyclinA/cyclin D1/CDK4/p21	proliferation (+); migration (+); invasion (+); cell cycle (+)	76
hsa_circ_101280	SLAIN1	Up	miR-375	JAK2	proliferation (+); apoptosis (-)	77
circRNA-101368		Up	miR-200a	HMGB1/RAGE/NF-κB/E-Cadherin	migration (+)	78
circ-ZEB1.33		Up	miR-200a-3p	CDK6		79
circFBLIM1	FBLIM1	Up	miR-346	FBLIM1	proliferation (+); invasion (+); apoptosis (-)	80
hsa_circ_0103809	AP4E1	Up	miR-490-5p	SOX2	proliferation (+); migration (+); apoptosis (-)	81
hsa_circ_0016788	TRIM11	Up	miR-486	CDK4	proliferation (+); invasion (+); apoptosis (-)	82
hsa_circRBM23	RBM23	Up	miR-138	vimentin/CCND3	viability (+); proliferation (+); migration (+); cell cycle (+)	83
hsa_circ_0005075	EIF4G3	Up	miR-431		proliferation (+); migration (+); invasion (+)	84
hsa_circ_0067934	PRKC1	Up	miR-1324	FZD5/Wnt/β-catenin	proliferation (+); migration (+); invasion (+); apoptosis (-)	85
circABCC2	ABCC2	Up	miR-665	ABCC2	proliferation (+); invasion(+)	70
hsa_circ_100338	SNX27	Up	miR-141-3p	ZEB1	proliferation (+)	86
CircFBXO11	FBXO11	Up	miR-605	FOXO3/ABCB1	proliferation (+); cell cycle (+); chemotherapy; resistance (+)	87
circ_0091581		Up	miR-526b	c-MYC	proliferation (+)	88
circPCNX	PCNX	Up	miR-506	Snail2/YAP	proliferation (+); apoptosis (-)	89
Circ-PRMT5	PRMT5	Up	miR-188-5p	HK2	proliferation (+); migration (+); glycolysis (+)	90
hsa_circ_0000092		Up	miR-338-3p	HN1	proliferation (+); migration (+); invasion (+); apoptosis (-)	91
hsa_circ_0056836		Up	miR-766-3p	FOSL2	proliferation (+); migration (+); invasion (+)	92
circ- HOMER1	HOMER1	Up	miR-1322	CXCL6	proliferation (+); migration (+); invasion (+); apoptosis (-)	93
CircABCB10	ABCB10	Up	miR-670-3p	HMG20A	proliferation (+); invasion (+)	94
circ_0091579		Up	miRNA-490-3p		proliferation (+); migration (+)	95
circ_0001955		Up	miR-516a-5p	TRAF6/MAPK11	proliferation (+)	96
Circ-TCF4.85		Up	miR-486-5p	ABCF2	proliferation (+); migration (+); invasion (+); apoptosis (-)	97

**Table 2:** The down-regulated circRNAs in HCC

CircRNA	Gene Symbol	Expression Change	miRNA sponged	Competitive mRNA	Function	Reference
cSMARCA5	SMARCA5	Down	miR-17-3p; miR-181b-5p	TIMP3	proliferation (-); migration (-)	38
circTRIM33-12	TRIM33	Down	miR-191	TET1/5hmC	proliferation (-); migration (-); invasion (-); immune evasion (-)	98
circMTO1	MTO1	Down	miR-9	p21	Proliferation (-); invasion (-)	99
circADAMTS13	ADAMTS13	Down	miR-484		Proliferation (-);	100
circADAMTS14	ADAMTS14	Down	miR‐572	RCAN1	proliferation (-); migration (-); invasion (-); apoptosis (+)	11
hsa_circ_0070269	PLAC8	Down	miR-182	NPTX1	proliferation (-); invasion (-)	101
circRNA_101505		Down	miR-103	NOR1	proliferation (-); apoptosis (+); cisplatin resistance (-)	102
hsa_circ_0091570	MBNL3	Down	miR-1307	ISM1	proliferation (-); migration (-); apoptosis (+)	103
hsa_circ_0001649	SHPRH	Down	miR-127-5p; miR-612; miR-4688	SHPRH	proliferation (-); migration (-)	104
circSETD3	SETD3	Down	miR-421	MAPK14	proliferation (-); cell cycle (-); migration (-)	105
circSMAD2	SMAD2	Down	miR-629	EMT	migration (-); invasion (-); EMT (-)	106
circC3P1	C3P1	Down	miR-4641	PCK1	proliferation (-); migration (-); invasion (-)	107
circ-ABCB10	ABCB10	Down	miR-340-5p; miR-452-5p	NRP1; ABL2	proliferation (-); migration (-); invasion (-); apoptosis (+)	108
CircRNA-0072309		Down	miR-665	PI3K/AKT and Wnt/β-catenin pathways	proliferation (-); migration (-); invasion (-); apoptosis (+)	109
circ-0051443		Down	miR-331-3p	BAK1	cell cycle (-); apoptosis (+)	110
circRNA-5692		Down	miR-328-5p	DAB2IP	proliferation (-); migration (-); invasion (-); apoptosis (+)	12

## Abbreviations

HCC: hepatocellular carcinoma
TACE: transcatheter arterial chemoembolization
CircRNAs: circular RNAs
EcircRNAs: exon circRNAs
CiRNAs: circular intron RNAs
EIciRNAs: exon-intron circRNAs
ncRNA: non-coding RNA
RBPs: RNA-binding proteins
miRNAs: microRNAs
MREs: miRNA response element
CDR1as: cerebellar Degeneration-Related protein 1 antisense
HuR: human antigen R
IRES: internal ribosome entry sites
ncRNAs: non-coding RNAs
EMT: epithelial-mesenchymal transition
